# Correlation of a new hydrodynamic index with other effective indexes in Chiari I malformation patients with different associations

**DOI:** 10.1038/s41598-020-72961-0

**Published:** 2020-09-28

**Authors:** Seifollah Gholampour, Hanie Gholampour

**Affiliations:** 1 Department of Biomedical Engineering, North Tehran Branch, Islamic Azad University, Tehran, Iran; 2 Department of Electrical and Computer Engineering, Science and Research Branch, Islamic Azad University, Tehran, Iran

**Keywords:** Computational biophysics, Biomedical engineering, Mechanical engineering, Biophysical models

## Abstract

This study aimed to find a new CSF hydrodynamic index to assess Chiari type I malformation (CM-I) patients’ conditions and examine the relationship of this new index with morphometric and volumetric changes in these patients and their clinical symptoms. To this end, 58 CM-I patients in four groups and 20 healthy subjects underwent PC-MRI. Ten morphometric and three volumetric parameters were calculated. The CSF hydrodynamic parameters were also analyzed through computational fluid dynamic (CFD) simulation. The maximum CSF pressure was identified as a new hydrodynamic parameter to assess the CM-I patients’ conditions. This parameter was similar in patients with the same symptoms regardless of the group to which they belonged. The result showed a weak correlation between the maximum CSF pressure and the morphometric parameters in the patients. Among the volumetric parameters, PCF volume had the highest correlation with the maximum CSF pressure, which its value being higher in patients with CM-I/SM/scoliosis (R^2^ = 65.6%, P = 0.0022) than in the other patients. PCF volume was the more relevant volumetric parameter to assess the patients’ symptoms. The values of PCF volume were greater in patients that headache symptom was more obvious than other symptoms, as compared to the other patients.

## Introduction

Investigation of Chiari type I malformation (CM-I), among various malformations of the craniocervical junction, is of great significance because of its prevalence (greater than 3%) and the severity of its symptoms^1^. CM-I is a clinical syndrome defined as a notable herniation of the cerebellar tonsils (greater than 5 mm) through the foramen magnum^2–4^. Neural/dural structures in the craniocervical junction of patients suffering from CM-I often undergo compression with appearance of some clinical symptoms in the patients. Hence, symptom assessment is highly critical in these patients^5^. These clinical symptoms are used in many studies as an index for assessing patients’ health conditions^5–7^. Hwever, it is difficult to diagnose, manage, and choose the appropriate treatment strategy for the patients only based on their symptoms, since many symptoms of this disease are often associated with pain and are common in the various associations and conditions of CM-I^8,9^. Furthermore, previous studies have shown that CM-I patients with a small tonsillar herniation may have severe clinical symptoms whereas patients with a large tonsillar herniation may show no symptoms^10,11^. Hence, despite the progress in this area, there is still little information available about symptoms related to this disease^4^, making its diagnosis difficult.

The primary diagnosis of this disease is achieved generally with neuroimaging techniques. The reason is that the incidence of CM-I is usually accompanied by obvious morphological and volumetric changes in patients^8,12^. Accordingly, several studies have used morphological and volumetric changes in patients as a diagnostic parameter and an index for investigating the severity of the disease and/or efficacy of surgery^13–18^. In recent years, numerous studies have been performed to obtain new morphometric and volumetric parameters in addition to tonsilar herniation for diagnosis and treatment of CM-I patients. Some studies have investigated the relationship between different morphometric and volumetric parameters affecting this disease or the relationship between these parameters and patients’ symptoms^2,19^. Despite all these efforts and advances in neuroimaging technologies, there are still different and sometimes conflicting opinions about which morphological and volumetric parameters are more effective and relevant than other parameters^12,17,20–28^. For example, some studies have considered the reduction of posterior cranial fossa (PCF) volume as the primary characteristic of CM-I^17,29,30^. However, some studies have found no association between PCF volume and the incidence of CM-I^20,31,32^. Therefore, previous studies on morphological and volumetric parameters affecting CM-I are not sufficiently comprehensive to diagnose this disease, and there are also many contradictions in these groups of studies.

However, another group of studies has shown that the role of cerebrospinal fluid (CSF) hydrodynamics is highly prominent in managing many diseases associated with the CSF circulation system^21,33–38^. Hydrodynamic studies of CSF have shown that the incidence of CM-I is accompanied by changes in hydrodynamic parameters of CSF flow^21,39–46^. Many studies have compared CSF velocity changes measured with phase contrast magnetic resonance imaging (PC-MRI) to assess patients’ conditions or outcomes of surgical procedures^47–50^. However, PC-MRI has limitations in measuring velocity distribution at all locations and under all conditions of patients, and further, it cannot measure CSF pressure^51,52^. Hence, CSF hydrodynamic parameters have been calculated in many studies non-invasively using computational fluid dynamic (CFD) simulation and have been used as an index for assessing conditions and surgical outcomes of CM-I patients^21,53–58^. Despite numerous studies performed for assessing hydrodynamic parameters affecting CM-I, many studies still doubt the role of CSF hydrodynamics in the pathophysiology of CM-I patients^53,59^, and thus, are uncertain about the significance of hydrodynamic parameters.

Findings about the diagnosis and treatment of CM-I, pathophysiology of the disease, prognosis of the disease, severity of the symptoms, and surgical outcomes of CM-I patients are still conflicting, controversial, and unclear^40,60,61^. The hypothesis in the present study is that a new hydrodynamic index may be defined that can respond to the conflicts emerged about the assessment of CM-I patients. Attempt was made to examine the interrelationship of this hydrodynamic index with clinical symptoms as well as morphometric and volumetric parameters of CM-I patients with different associations.

## Method

### Study population

Many studies have pointed out the significance of different conditions and associations of CM-I during the diagnosis and treatment processes of patients^62,63^. Previous studies have shown that the exact pathogenesis of syringomyelia (SM) as the most common association of CM-I is unclear. The association of scoliosis in adult CM-I patients has not also been studied thoroughly and comprehensively^24,48,64–66^. Therefore, CM-I patients with SM (CM-I/SM) as well as CM-I patients with SM and scoliosis (CM-I/SM/scoliosis) were classified into two separated groups, in addition to CM-I patients in the present study. Since less attention has been paid to the communication of CM-I associations, such as tethered cord syndrome (TCS)^21,67^, CM-I patients with TCS (CM-I/TCS) were classified in another group for assessment and comparison with the other groups. Accordingly, briefly, patients recruited for this study were classified into four general groups: 19 patients suffering from CM-I (age = 29–54 years, BMI = 22.9 ± 0.6, 63% female), 14 patients with CM-I/TCS (age = 26–55 years, BMI = 23.4 ± 0.7, 56% female), 15 patients with CM-I/SM (age = 27–49 years, BMI = 23.0 ± 0.7, 56% female), and 10 patients with CM-I/SM/scoliosis (age = 25–49 years, BMI = 23.2 ± 0.8, 60% female). Furthermore, to compare the patients’ conditions with healthy conditions, 20 healthy subjects (age = 26–57 years, BMI = 23.3 ± 0.8, 60% female) were recruited.

It should be noted that samples in this research were selected based on the diagnosis and confirmation of the neurosurgery team at the Shohada Tajrish Hospital from adult CM-I patients admitted to this hospital. It is noteworthy that the healthy subjects in this study had no history of CM-I or associated disorders and surgeries, and none of the patients had a history of TCS surgeries, scoliosis surgeries, and other Chiari-related surgeries. According to the diagnosis of the neurosurgery team, general symptoms of the patients included headache (84%), severe pain (68%), sensory disturbances (51%), nausea/vomiting (40%), weakness (33%), urinary dysfunction (28%), and cranial nerve deficits (18%), with the first three being the most prevalent symptoms. Hence, the first three clinical symptoms were only assessed in this study. It should be noted that “pain” in the study refers to neck and back pains.

All procedures and methods performed in studies involving human participants were conducted in accordance with the Declaration of Helsinki 1964 and its later amendments. The study design and protocol were approved by the Ethics Committee of the Functional Neurosurgery Center at the Shohada Tajrish Hospital with the ethics number 17/59-1. Prior to scanning, written informed consent was obtained from all the volunteers. All MRI data were anonymized prior to transfer to operators for analysis.

### MR imaging data, in vivo measurements, and 3D geometric modeling

MRI files were obtained from the 58 patients and the 20 healthy subjects. The samples lied in the supine position on the scanner bed with an MR imaging (Magnetom Trio; Siemens, Erlangen, Germany), which included localizer, sagittal T1-weighted (field of view (FOV): 16 cm, matrix: 256 × 256, section thickness: 3 mm, NEX: 3, and TR/TE: 520/10) and sagittal T2-weighted (FOV: 16 cm, matrix: 512 × 512, section thickness: 3 mm, and TR/TE: 3600/116) images. The process of separating the subarachnoid space, spinal cord, and cerebellum for each sample was carried out using the high contrast of CSF surrounding tissues with CSF flow. PC-MRI scans were performed at the craniospinal junction (C1) for using as inlet boundary conditions and at the C4 level for using in the data validation process. By using an axial scan plane, velocity-encoded gradient-echo imaging (Venc) was aligned normal to CSF flow. Acquisition parameters for phase-contrast flow measurement with axial PCMR were: FOV: 180 mm, matrix: 256 × 256, section thickness: 5 mm, TR/TE: 20/5 ms, flip angle: 20°, and Venc = 9–13 cm/s. A set of 3D images was obtained by 10,000 projections of a volume of 20 × 20 × 20 cm^3^ and with 256 × 256 × 256 voxels during 5 min in each sample. Details of the standard method of MRI preparation are accessible in studies by Rutkowska et al. and Stroman et al.^45,68^.

**Figure 1:**
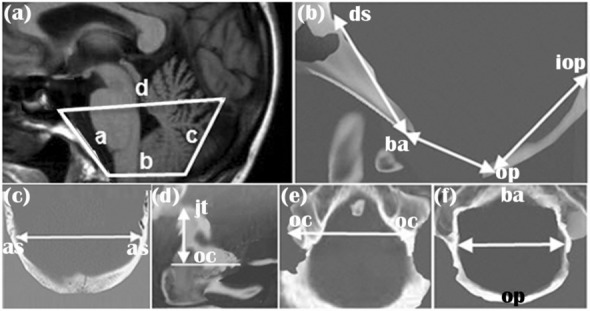
Panels show the measurements of PCF made on T1-weighted MRI. **(a)** a: clivus; b: foramen magnum; c: supraocciput; d: Twining line. **(b)** Distance from the opisthion (op) to the inner surfaces of the basion (ba) is the anterior–posterior diameter of the foramen magnum; distance from the opisthion to the center of the internal occipital protuberance (iop) is the axial length of supraocciput; and distance from the basion to the top of the dorsum sellae (ds) is the axial length of clivus. **(c)** Shown the distance between asterions (as). **(d)** The perpendicular distance from the top of the jugular tubercle (jt) and the bottom of the occipital condyle (oc) along a line parallel with the orbitomeatal line is the axial height of condyle. **(e)** Distance between outer surfaces of occipital condyles (oc) is the width of condyles. **(f)** Distance between opisthion (op) and the basion (ba) is the maximum transverse diameter of the foramen magnum.

The data obtained from PC-MRI yielded three outputs. The first output was the DICOM file of each sample, which was transferred to Mimics software (Materialise, Ann Arbor, Mich) for segmenting the subarachnoid space, spinal cord, and cerebellum manually. A snapshot of the images was prepared at each cervical level. The desirable region was selected, and intensities of that signal range were determined. All voxels in the signal intensity range were highlighted, and the upper and lower threshold values were adjusted iteratively for displaying their voxels. In the direction of the axis, the outlet and inlet of the canal were cropped at each end. Based on a cloud of points obtained from Mimics, 3D geometric models of the samples were created using CATIA version 5R21 software (Dassault Systems; Waltham, MA, USA) through fitting the surfaces on the points. The surfaces of the geometric models were smoothed manually using this software. A 3D geometric model of CSF volume was created by geometric modeling of the internal layers of the spinal subarachnoid space. It should be noted that all rendering processes of the 3D models were carried out using CATIA software. Thereafter, 11 morphometric parameters were measured for the 58 patients and the 20 healthy subjects using CATIA (Fig. 1 and Table 1). Three volumetric parameters including the posterior fossa brain (PFB) volume, CSF volume, and PCF volume were also measured using CATIA for all the patients and healthy subjects (Table 1). The 3D geometric models of the samples were then transferred to ABAQUS version 6.14 (Dassault Systems; Waltham, MA, USA) for meshing. The CSF hydrodynamic parameters of the samples were calculated following CFD simulation using ABAQUS.

**Table 1:** Analysis of morphometric parameters, volumetric parameters and CSF hydrodynamic parameters. The value of each parameter is its mean value in the samples of that group. It should be noted that the percentage of CV is listed in this table.

Parameters						Normal	CM-I	CM-I/TCS	CM-I/SM	CM-I/SM/scoliosis
Morphometric parameters	Occipital bone size	Foramen magnum	Transverse diameter (mm)		Mean ± confidence value	31.6 ± 1.8	27.5 ± 1.6	35.8 ± 2.1	28.1 ± 1.6	34.7 ± 2.5
					SD/CV	4.1/12.9	3.6/13.1	4.1/11.5	3.1/11.0	4.0/11.5
			Anterior–posterior diameter (mm)		Mean ± confidence value	33.6 ± 2.0	33.0 ± 1.8	37.2 ± 2.2	33.9 ± 2.1	35.8 ± 2.5
					SD/CV	4.2/12.5	4.1/12.4	4.2/11.3	4.1/12.1	4.1/11.4
		Supraocciput	Distance between asterions (mm)		Mean ± confidence value	105.7 ± 5.4	98.6 ± 5.3	106.5 ± 6.3	104.9 ± 5.5	105.4 ± 6.7
					SD/CV	12.3/11.6	11.9/12.0	12.0/11.3	10.9/10.4	10.8/10.2
			Axial length (mm)		Mean ± confidence value	48.2 ± 2.5	40.3 ± 2.1	47.3 ± 2.6	48.0 ± 2.5	46.3 ± 3.0
					SD/CV	5.6/11.6	4.7/11.7	4.9/10.4	4.9/10.2	4.8/10.4
		Clivus	Distance between carotid canals (mm)		Mean ± confidence value	20.9 ± 1.2	17.8 ± 0.9	21.1 ± 1.2	21.0 ± 1.5	21.0 ± 1.8
					SD/CV	2.8/13.4	2.1/11.8	2.4/11.4	2.9/13.8	2.9/13.8
			Axial length (mm)		Mean ± confidence value	47.9 ± 2.5	39.6 ± 2.1	46.4 ± 2.9	47.4 ± 3.0	45.8 ± 3.6
					SD/CV	5.6/11.7	4.8/12.1	5.5/11.8	5.9/12.4	5.8/12.7
		Condyle	Width of condyles (mm)		Mean ± confidence value	51.8 ± 2.8	45.9 ± 2.2	57.9 ± 3.1	51.0 ± 3.1	56.2 ± 3.8
					SD/CV	6.4/12.4	5.0/10.9	6.0/10.4	6.1/11.3	6.1/10.5
			Axial height right (mm)		Mean ± confidence value	25.2 ± 1.4	18.0 ± 1.0	24.5 ± 1.6	26.8 ± 1.6	23.9 ± 1.9
					SD/CV	3.2/12.7	2.2/12.2	3.1/12.6	3.2/11.9	3.1/12.9
			Axial height left (mm)		Mean ± confidence value	25.9 ± 1.4	19.2 ± 1.0	25.7 ± 1.6	27.4 ± 1.6	24.0 ± 1.9
					SD/CV	3.2/12.4	2.2/11.4	3.1/12.0	3.2/11.7	3.1/12.9
	Size of brain structures		Axial length of the brain stem (mm)		Mean ± confidence value	52.2 ± 4.1	53.6 ± 4.1	57.4 ± 4.8	58.3 ± 4.7	57.4 ± 5.8
					SD/CV	9.3/17.8	9.2/17.1	9.2/16.0	9.3/15.9	9.3/16.2
Volumetric parameters	PFB volume (ml)			Mean ± confidence value		164.4 ± 7.1	161.8 ± 7.2	163.6 ± 8.4	163.0 ± 8.2	162.3 ± 9.9
				SD/CV		16.2/9.8	16.1/9.9	16.0/9.8	16.2/9.9	16.1/9.9
	CSF volume (ml)			Mean ± confidence value		37.6 ± 1.7	16.4 ± 0.8	34.2 ± 1.9	17.8 ± 0.9	36.8 ± 2.4
				SD/CV		3.9/10.4	1.8/11.0	3.7/10.8	1.8/10.1	3.9/10.6
	PCF volume (ml)			Mean ± confidence value		209.2 ± 5.2	185.1 ± 5.4	201.6 ± 5.8	179.8 ± 5.9	167.7 ± 5.5
				SD/CV		11.8/5.6	12.1/6.5	11.1/5.5	11.6/6.4	8.9/5.3
CSF hydrodynamic parameters	Maximum systolic velocity (cm/s)			Mean ± confidence value		4.92 ± 1.1	5.25 ± 1.1	5.20 ± 1.1	5.31 ± 1.2	5.12 ± 0.7
				SD/CV		2.4/48.7	2.5/47.6	2.1/40.4	2.3/43.3	1.1/21.5
	Maximum diastolic velocity (cm/s)			Mean ± confidence value		4.68 ± 1.0	4.97 ± 1.1	5.01 ± 1.1	5.12 ± 1.2	4.93 ± 0.7
				SD/CV		2.3/49.1	2.4/48.3	2.1/41.9	2.3/44.9	1.1/22.3
	Maximum Reynolds number			Mean ± confidence value		711 ± 7.5	749 ± 7.2	734 ± 7.8	783 ± 8.7	760 ± 7.4
				SD/CV		17.2/2.4	16.1/2.1	15.0/2.0	17.2/2.2	12.0/1.6
	Maximum systolic pressure (cm H_2_O)			Mean ± confidence value		18.13 ± 0.7	22.89 ± 0.9	22.46 ± 1.0	22.32 ± 1.0	25.06 ± 1.4
				SD/CV		1.6/8.8	2.0/8.7	2.0/8.9	2.0/8.9	2.2/8.8
	Maximum diastolic pressure (cm H_2_O)			Mean ± confidence value		18.41 ± 0.6	23.16 ± 0.9	22.90 ± 1.0	22.70 ± 1.0	26.66 ± 1.4
				SD/CV		1.5/8.1	2.0/8.6	2.0/8.7	2.0/8.8	2.2/8.2

*CM-I* Chiari malformation type I, *TCS* tethered cord syndrome, *SM* syringomyelia, *PFB* posterior fossa brain, *CSF* cerebrospinal fluid, *PCF* posterior cranial fossa, *SD* standard deviation, *CV* coefficient of variation.

The second PC-MRI output was the CSF velocity of each sample at the craniospinal junction (C1), which was used as an inlet boundary condition during the simulation process. The third PC-MRI output was the CSF velocity graph at the C4 level of each sample. This graph was used for validating the CFD simulation results.

### Computer simulation

Fluid–structure interaction (FSI) and CFD methods were used in previous studies for analyzing CSF hydrodynamics in the spinal cord. In reality, the spinal subarachnoid space appears to be a deformable boundary, as shown by Tangen et al.^69–71^, and hence, the FSI method can be considered as a representative for this deformable and movable boundary. However, this deformation was neglected in the majority of previous studies assessing CSF hydrodynamics in CM-I patients. They chose no-slip boundary conditions at the spinal cord and dural boundaries, and used the CFD solution method for simulations^21,43,45,46,53–56,72,73^. Therefore, according to the main focus of the present study and based on the results of previous studies, the spinal subarachnoid space is defined as a non-deformable boundary and CSF is considered as an incompressible Newtonian fluid^21,46,54,55,72,73^ for the CFD simulation of CSF hydrodynamics in normal subjects and patients. Equations governing the fluid were conservation of mass and conservation of momentum according to Eqs. (1) and (2), respectively ^74–78^:

1 $$\overrightarrow{\nabla }.\left(\uprho \overrightarrow{u}\right)=0$$

2 $$\frac{\partial \overrightarrow{u}}{\partial t}+\overrightarrow{u}.\nabla \overrightarrow{u}=-\frac{1}{\rho }\overrightarrow{\nabla }\mathrm{p}+v\overrightarrow{{\nabla }^{2}}+\overrightarrow{F}$$

where $\rho$,$\overrightarrow{F}$, P, $\overrightarrow{u}$, and $v$ are the density, external force, pressure fields, periodic velocity, and kinematic viscosity of CSF, respectively.

It should be noted that the CSF density and kinematic viscosity were defined as 1000 kg/m^3^ and 0.7 × 10^–6^ m^2^/s, respectively ^21,52,56^. The in vivo pulsatile CSF flow at the craniospinal junction (C1) (the second PC-MRI output) was used as the inlet boundary condition, which was also used in many previous studies such as the study by Yiallourou et al. ^79^. According to previous studies, the zero-pressure was defined as the outlet boundary condition ^21,43,53,55,72^.

**Figure 2:**
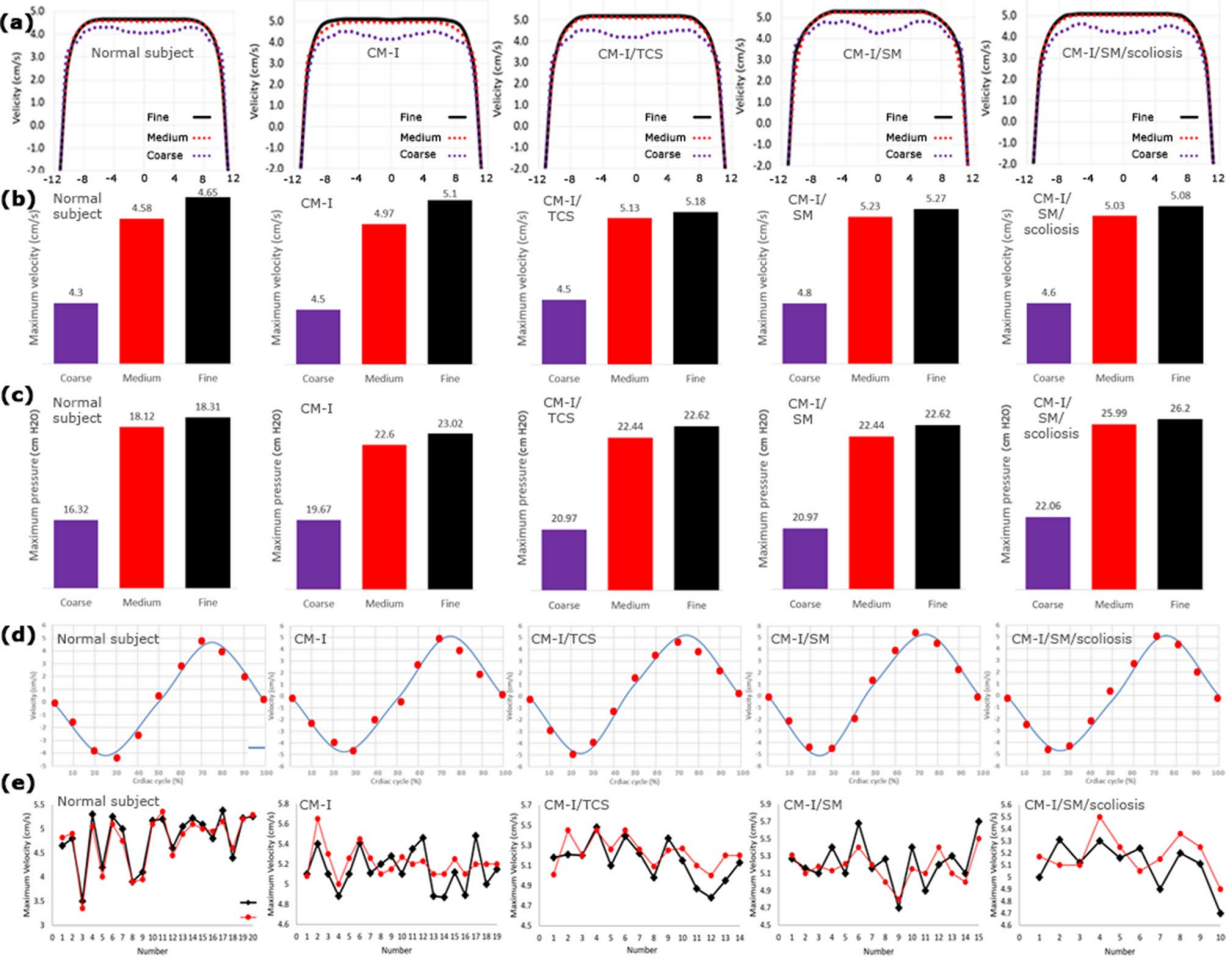
The mesh convergence study for the three grids: coarse, medium and fine meshes: **(a)** velocity profile along with line K (line K is shown in Fig. 3b), **(b)** maximum systolic velocity, **(c)** maximum systolic pressure. **(d)** The comparison of the PC-MRI velocity graph and the velocity graph calculated by the CFD simulation systolic. **(e)** The comparison of the maximum systolic velocity obtains from PC-MRI and CFD simulation for all samples.

**Figure 3:**
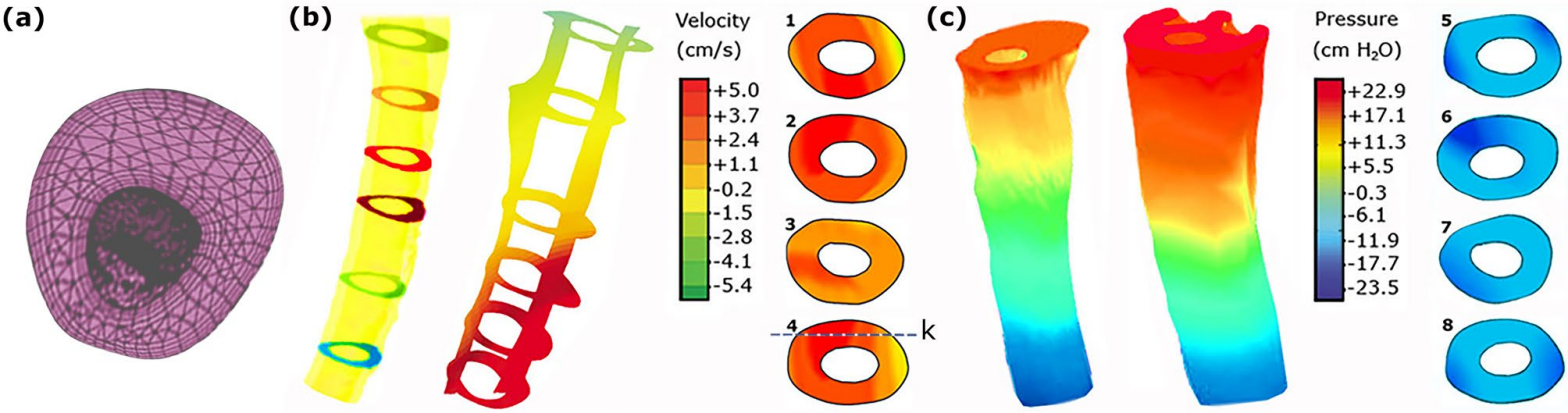
**(a)** The type and size of meshes near the walls were defined differently from other locations for considering the effect of the boundary layer. **(b)**, **(c)** compare the velocity and pressure distributions, respectively. (1), (2), (3), and (4) are the velocity distribution at the level of C5 for patients number 1 in CM-I 1, CM-I/SM, CM-I/hydrocephalus, and CM-I/TCS, respectively. (5), (6), (7), and (8) are the pressure distribution at the level of C6 for similar patients. The left side models of velocity and pressure distribution in panels **(c)** and **(d)** belong to the CM-I patient number 1. K is the direction of the assessment of velocity if Fig. 2a for the mesh convergence study.

CSF pressure and velocity were calculated at discretized representations (meshes) at each time step (0.001 s) and computational points by solving Eqs. (1) and (2) for each sample. It should be noted that the iteration number during CFD simulation was equal to 50. The mesh convergence study was conducted by analyzing the effect of mesh density on the maximum CSF pressure and velocity. The maximum differences between the CSF results (velocities and pressures) in the medium and fine meshes in all the samples were less than 2.55% (Fig. 2a–c). This acceptable difference ensured mesh convergence in terms of mesh density. The number of meshes obtained for the patient number 1 in CM-I, CM-I/TCS, CM-I/SM, and CM-I/SM/scoliosis groups and the healthy subject number 1 were 832,100, 809,158, 848,610, 853,062, and 716,953, respectively. To consider the effect of the boundary layer during the mesh generation, the type and size of meshes near the walls were defined differently from other locations (Fig. 3a). It should be noted that it took about three years to complete full modeling and analysis process and reach the mesh convergence for the results of all healthy subjects and patients using a CPU with a 16-core server processor.

### Statistics

Mean, confidence value, standard deviation (SD) and coefficient of variation (CV), which is the ratio of the SD to the mean (SD/mean), were calculated for hydrodynamic, volumetric, and morphometric parameters in each group of patients and healthy subjects using SPSS software version 20. It should be noted that the results of the Kolmogorov–Smirnov test and Pearson’s correlation coefficient (PCC) were also calculated in SPSS software. The p-value of 0.05 was considered statistically significant (original α-value = 0.05).

## Results

### Data validation

Data validation is one of the most significant concerns in computer simulation studies. Comparison of CSF velocity data measured in vivo using PC-MRI with CSF velocity data calculated by biomechanical simulation is one of the methods used for validating assumptions, inputs and outputs, boundary conditions, and applied solution in previous computer simulation studies^21,51,52,72,80–82^. Therefore, CSF velocity at the level of C4 was compared for all the healthy subjects and patients using the experimental method of PC-MRI, and CFD simulation (Fig. 2d,e). The reason that CSF velocity was compared, specifically at the C4 level, with these two methods in each sample was that the maximum CSF velocity occurred between the C3–C5 level in all the healthy subjects and patients.

The phase difference between the two velocity graphs was less than 0.1% for all the samples (Fig. 2d). It should be noted that the maximum difference between the maximum CSF velocity data obtained from the two above-mentioned methods was less than 5.1% in all the samples, as shown in Fig. 2e. The comparisons indicated an acceptable agreement between these two groups of velocity data, and this agreement ensured the correctness of the software solution process. All pressure and velocity values listed in Table 1 were the results of CFD simulation.

### CSF hydrodynamic parameters

**Figure 4:**
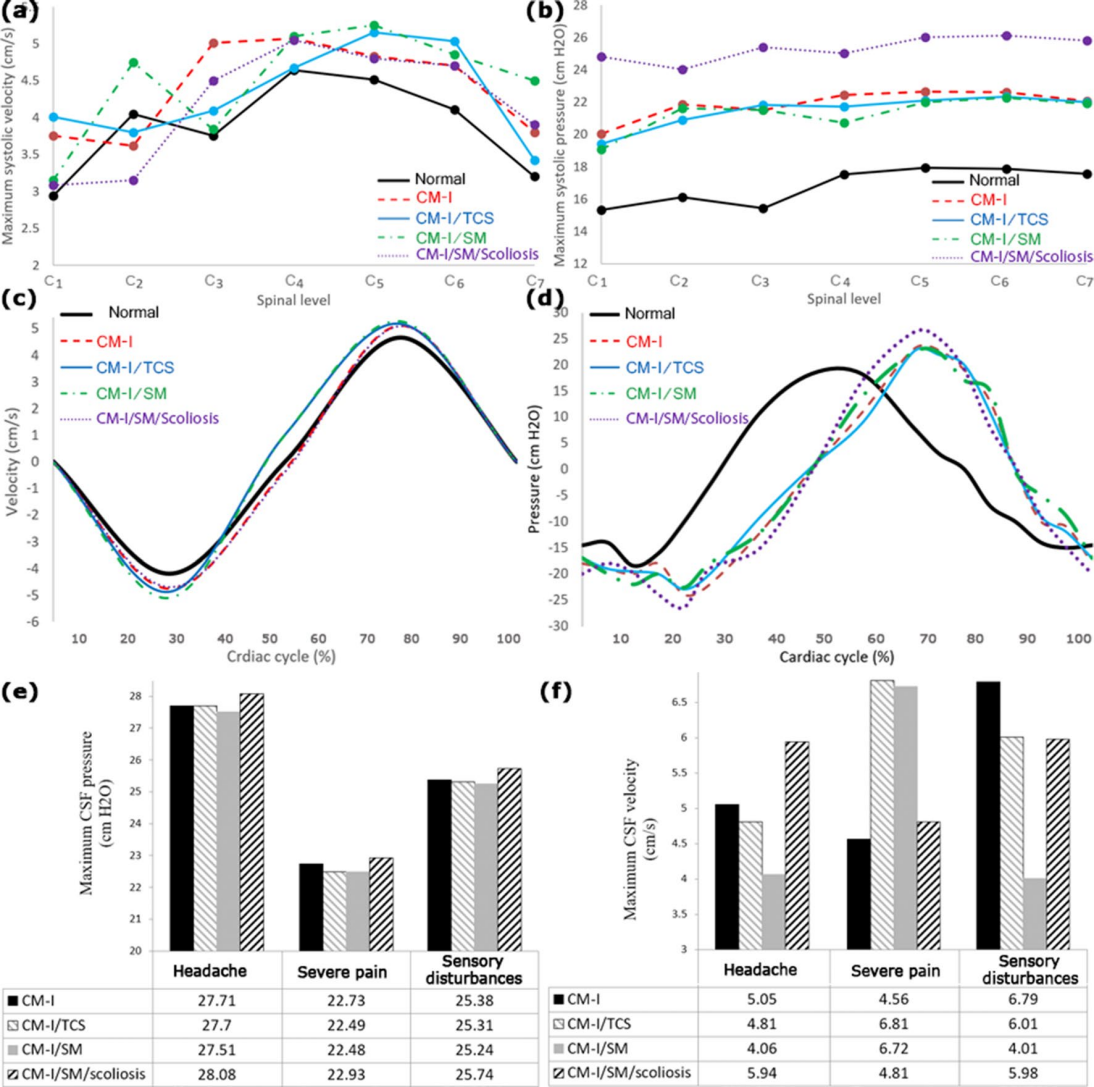
**(a)**, **(b)** the maximum CSF velocity and pressure in the term of spinal level for healthy subject number 1 and patients number 1, respectively. **(c)**, **(d)** compare the CSF velocity and pressure graphs of healthy subject number 1 and patients number 1, respectively. **(e)**, **(f)** show the relationship between prevalent symptoms of patients with maximum CSF pressure and velocity, respectively. For example, the intensity of the “headache” symptom was higher and more obvious than that of the other symptoms in the patients.

All computer calculations were performed for five cardiac cycles in each sample. As there were no discrepancies in the results of the 4th and 5th cycle, the results of the 5th cycle were reported in this section. The distribution of CSF velocity and pressure in the healthy subjects and patients are shown in Fig. 3b,c, respectively. The results showed that the location of the maximum CSF velocity was at the C3–C5 level, and that the maximum CSF pressure occurred at the C5–C6 level (Fig. 4a,b). The graphs of CSF velocity in the healthy subjects and patients were compared during a cardiac cycle, as shown in Fig. 4c. There was almost no phase difference between these graphs, and the only difference was in the amounts of the maximum CSF velocity. The velocity curves showed that CSF flow was mainly in the caudal direction during the systolic phase of the cardiac cycle and in the cranial direction during the diastolic phase of the cardiac cycle. According to the “CSF pressure-cardiac cycle” graphs in Fig. 4d, the highest pressure gradient occurs when the flow direction changes. It is commonly believed that a pressure gradient across the cranial and spinal CSF compartments leads to these diseases. This pressure gradient is mainly due to raised cranial pressure or fluid flow diversion. According to Table 1, the maximum CSF systolic and diastolic velocities increased by 4.1% to 9.4% in the patients compared to the healthy subjects. However, the difference between the amounts of the maximum CSF velocity was small in patients of different groups. The results of Table 1 also showed that the maximum CSF systolic pressure in CM-I, CM-I/TCS, CM-I/SM, and CM-I/SM/scoliosis patients was respectively 26.2%, 23.9%, 23.1%, and 38.2% greater than the maximum CSF systolic pressure in the healthy subjects. The corresponding values for the maximum CSF diastolic pressure were 25.8%, 24.4%, 23.3%, and 44.8%, respectively (Table 1). Further, in contrast to the velocity results, the maximum CSF pressure in CM-I/SM/scoliosis patients was at least 12.3% greater than that in the other three groups of patients.

Equation (3) yields the Reynolds number as an index for flow laminarity^75,83^:

3 $$\mathrm{Re}=\frac{\overrightarrow{u}d}{\nu }$$

where d is the diameter of the CSF path section and Re is the Reynolds number. Despite the difference (of less than 10.1%) of Reynolds numbers between the patients and healthy subjects (Table 1), the disease incidence did not result in the transition of the CSF flow regime from a laminar to a turbulent state.

### Morphometric and volumetric parameters

Assessment of morphological changes in CM-I patients is of great significance for predicting surgery risks and assessing patients’ conditions^8,12^, and craniospinal size changes are among the most obvious changes in these patients. Some studies have presented CSF volume changes during the incidence of CM-I as a highly prognostic and valuable parameter^84^. Studies have shown that examining changes in PFB and PCF volumes at the beginning of the disease has a notable diagnostic value^22–25,27,29,30^. Therefore, changes in the size of the occipital bone (foramen magnum, supraocciput, clivus, and condyle) and brain structures were considered as representatives of patients’ morphometric parameters in the present study (Fig. 1). The changes in the PFB, CSF, and PCF volumes of the samples were also examined as representatives of the volumetric parameters.

Among the morphometric parameters, the occipital bone size was lower in CM-I patients than in the healthy subjects according to Table 1. As shown in the table, the axial condylar height at the right and left sides experienced the highest reduction and decreased by 28.6% and 25.8%, respectively. In the other groups of patients, the difference of the occipital bone size was mostly insignificant between the patients and healthy subjects; the smallest difference (less than 1.0%) was related to the distance between the asterions of supraocciput and the distance between the carotid canals of clivus. The size of the brain structures had the highest increase in all the patients compared to the healthy subjects.

PFB volume in CM-I, CM-I/TCS, CM-I/SM, and CM-I/SM/scoliosis patients was respectively 1.6%, 0.5%, 0.9%, and 1.3% less than that in the healthy subjects (Table 1). Relevant values for CSF volume were 56.4%, 9.0%, 52.7%, and 2.1%, respectively (Table 1). Corresponding values for PCF volume were 11.5%, 3.6%, 14.0%, and 19.8%, respectively (Table 1). It should be noted that in the past, there was controversy about the difference of PCF volume between CM-I patients^17,20,29–32^, while Table 1 shows that the difference between CM-I/TCS and CM-I/SM/scoliosis was 20.2%. According to Table 1, CM-I/TCS disease resulted in the least change in PCF and PFB volumes, while CM-I/TCS and CM-I/SM/scoliosis diseases led to the least change in CSF volume. In all the three volumetric parameters, the PFB volume of the four groups of patients had the least change compared to the healthy subjects.

## Discussion

The first objective of this study was to introduce a more accurate and relevant CSF hydrodynamic index than hydrodynamic parameters introduced in previous studies to help with the assessment of CM-I patients. Further, the relationship of this index was investigated with symptoms of patients and with morphometric parameters including size of occipital bone (foramen magnum, supraocciput, clivus, and condyle) and brain structures as well as volumetric parameters including PFB, CSF, and PCF volumes. The aim was to evaluate the correlation level of each parameter for assessing conditions of CM-I patients and CM-I patients with three different associations.

According to the study by Rutkowska et al., Linge et al., and Roldan et al., the peak of CSF velocities was at the C4–C5, C3–C4 and C4 level, respectively^45,55,73^. In the present study, this peak value occurred at the C3–C5 level (Figs. 3b, 4a). The maximum systolic and diastolic velocities of CM-I patients were 5.25 ± 1.1 and 4.97 ± 1.1 (cm/s), respectively (Table 1). These values completely conform to studies by Linge et al.^54,73^. According to the studies by Linge et al., maximum Reynolds numbers were lower than 780 for CM-I patients^72^, which are close to the results of the present study (Table 1). The maximum systolic pressure of CM-I patients in the present study was 22.89 ± 0.9 (cm H_2_O) (Table 1), which conforms to the results of the study by Rutkowska et al.^45^.

It should be noted that it is not possible to calculate the absolute value of CSF pressure using CFD simulation and the Navier–Stokes equation, as only pressure gradient appears in terms of equations of fluid motion and only pressure differences relative to boundary pressures can be computed. Moreover, the behaviors of systolic and diastolic pressures or velocities were similar to each other during all the evaluations. Further, their correlations and relationships were completely similar to those of the other parameters. Therefore, we redefined the maximum CSF pressure/velocity in the present study as the relative peak pressure/velocity obtained from the normalizing function over the pressure/velocity wave based on systolic and diastolic peaks.

In this section, the relationships were investigated between the three most prevalent symptoms of the patients (headache, severe pain, and sensory disturbances) with the CSF velocity and pressure. It should be noted that in Fig. 4e,f, for example, the intensity of the “headache” symptom was higher and more obvious than that of the other symptoms in the patients. In all the groups, the maximum CSF pressure was greater in patients with the headache symptom than in patients with sensory disturbances (Fig. 4e). Further, the maximum CSF pressure was greater in patients with sensory disturbances than in patients with severe pain symptom (Fig. 4e). Therefore, there was a relationship between the maximum CSF pressure and the symptoms of patients in all the four groups. However, no relationship was observed between the maximum CSF velocities in different groups with similar symptoms (Fig. 4f). The results of Fig. 4e also showed that the difference between the values of the maximum CSF pressure in patients of different groups with similar symptoms was less than 2.0%. Therefore, the maximum CSF pressure was similar in patients with similar symptoms regardless of the group to which the patients belonged (Fig. 4e). The results also showed that the severity of these symptoms, despite their variety in the associations, was proportional to the maximum CSF pressure, and there were no considerable changes in the severity of symptoms in patients with similar maximum CSF pressure, even in different groups (Fig. 4e).

CV is the best index for comparison of data dispersion. It was found that the CV of the maximum CSF pressure data was less than 8.9% (Table 1). Under similar conditions and for similar samples, the CV of the maximum CSF velocity data was at least 2.4 times that of the maximum CSF pressure data, i.e. the maximum CSF velocity data were at least 2.4 times more dispersed (Table 1). Even in many samples, the maximum CSF velocity was higher in the healthy subjects than in the patients (Fig. 2e). According to the results of the relationship between the CSF hydrodynamic parameters and the symptoms of the patients, and comparison of data dispersion, it can be deduced that the maximum CSF pressure is a more accurate and relevant index than the maximum CSF velocity for assessing conditions of these patients.

Physicians establish the initial stages of management of these patients on the basis of examination of morphological and volumetrical changes in patients using MR images of their head and spine. Moreover, according to the above results, the maximum CSF pressure is a more accurate and relevant hydrodynamic parameter. Therefore, in this section, for all the four groups of patients, the correlation of the maximum CSF pressure with the morphometric parameters was evaluated in the first part, and its correlation with the volumetric parameters was evaluated in the second part. The results of the Kolmogorov–Smirnov test showed that the variables followed a normal distribution, and hence, we used PCC to assess the correlations. PCC of the maximum CSF pressure with the transverse diameter and the anterior–posterior diameters of foramen magnum in the four groups of patients were respectively 0.15–0.17 (P = 0.0035) and 0.15–0.17 (P = 0.0035). The corresponding PCC values for the distance between the asterions and axial length of supraocciput in the four groups of patients were respectively 0.11–0.13 (P = 0.0035) and 0.10–0.12 (P = 0.0035). The relevant PCC values for the distance between the carotid canals and axial length of clivus in the four groups of patients were 0.11–0.12 (P = 0.0035) and 0.10–0.13 (P = 0.0035). The corresponding PCC values for the width of condyles, axial height right, and axial height left of condyle in the four groups of patients were 0.11–0.13 (P = 0.0035), 0.11–0.13 (P = 0.0035), and 0.10–0.11 (P = 0.0035), respectively. The results also showed that PCC between the maximum CSF pressure and the axial length of the brain stem in the four groups of patients was 0.19–0.22 (P = 0.0035). These results showed that the correlation was extremely weak between the maximum CSF pressure and the morphometric parameters in all the four groups of patients.

**Figure 5:**
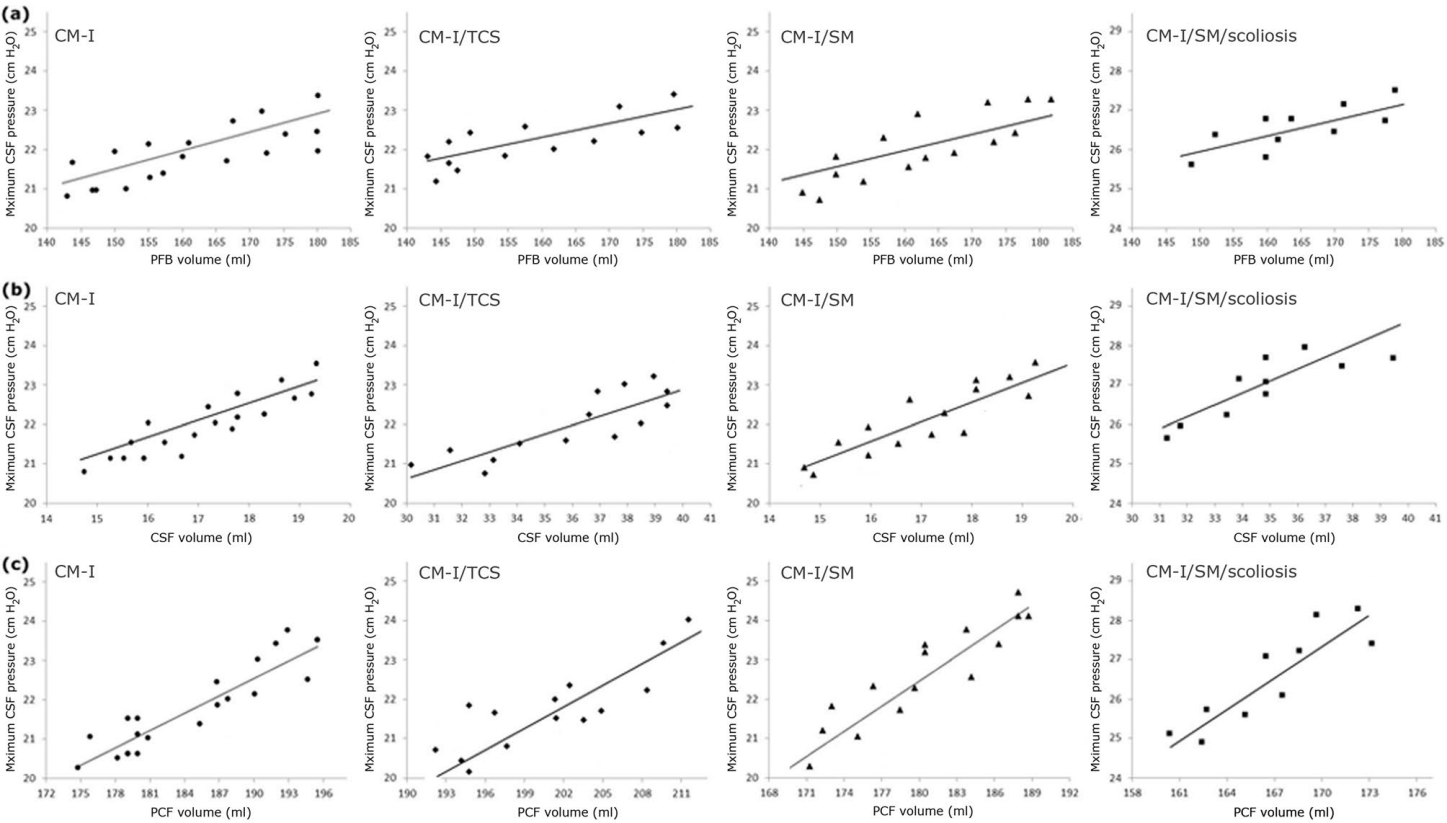
The PCC between the maximum CSF pressure of the patients with **(a)** PFB volumes, **(b)** CSF volumes, and **(c)** PCF volumes.

In this part, the correlation between the maximum CSF pressure and the volumetric parameters was investigated. The results showed that PCC between the maximum CSF pressure and PFB volume in patients suffering from CM-I, CM-I/TCS, CM-I/SM, and CM-I/SM/scoliosis was 0.39 (P = 0.0026), 0.28 (P = 0.0026), 0.32 (P = 0.0026), and 0.25 (P = 0.0026), respectively (Fig. 5a). The results of Fig. 5b showed that PCC between the maximum CSF pressure and CSF volume in CM-I, CM-I/TCS, CM-I/SM, and CM-I/SM/scoliosis patients was 0.45 (P = 0.0022), 0.44 (P = 0.0022), 0.46 (P = 0.0.0022), and 0.59 (P = 0.0022), respectively. The relevant values of PCC between the maximum CSF pressure and PCF volume were 0.67 (P = 0.0022), 0.72 (P = 0.0022), 0.75 (P = 0.0022), and 0.81 (P = 0.0022), respectively (Fig. 5c). The correlation of the maximum CSF pressure was higher with PCF volume than with other volumetric parameters. This correlation was also higher in patients with CM-I/SM/scoliosis (R^2^ = 65.6%, P = 0.0022) than in the other groups of patients. It should be noted that in the present study, there were multiple analyses on the same dependent variable of a single data set. Hence, it was necessary to assess the Bonferroni correction. The Bonferroni corrected P value equals the original α-value (0.05) divided by the number of dependent comparisons. In the present study. the maximum P value during calculations of all the aforementioned correlations was 0.0035. This means that the number of dependent comparisons needs to be less than 0.05/0.0035 = 14. In the present study, less than 14 dependent comparisons were assessed, and hence, the P values (0.0022–0.0035) were significant.

In addition to the correlation results, Table 1 shows that in the same samples and under the same conditions, the lowest CV, and subsequently, the lowest data dispersion among all the morphometric and volumetric parameters belonged to the PCF volume data, which was at least 1.6 times less than the CV of the other morphometric and volumetric parameters. Therefore, among the volumetric parameters, PCF volume is a more relevant parameter for assessing the conditions of these patients. This finding can also be useful in future studies for assessing the trend of changes in compliance (ΔV/ΔP) in these patients during their treatment process and for assessing their healing level^39,52^.

**Figure 6:**
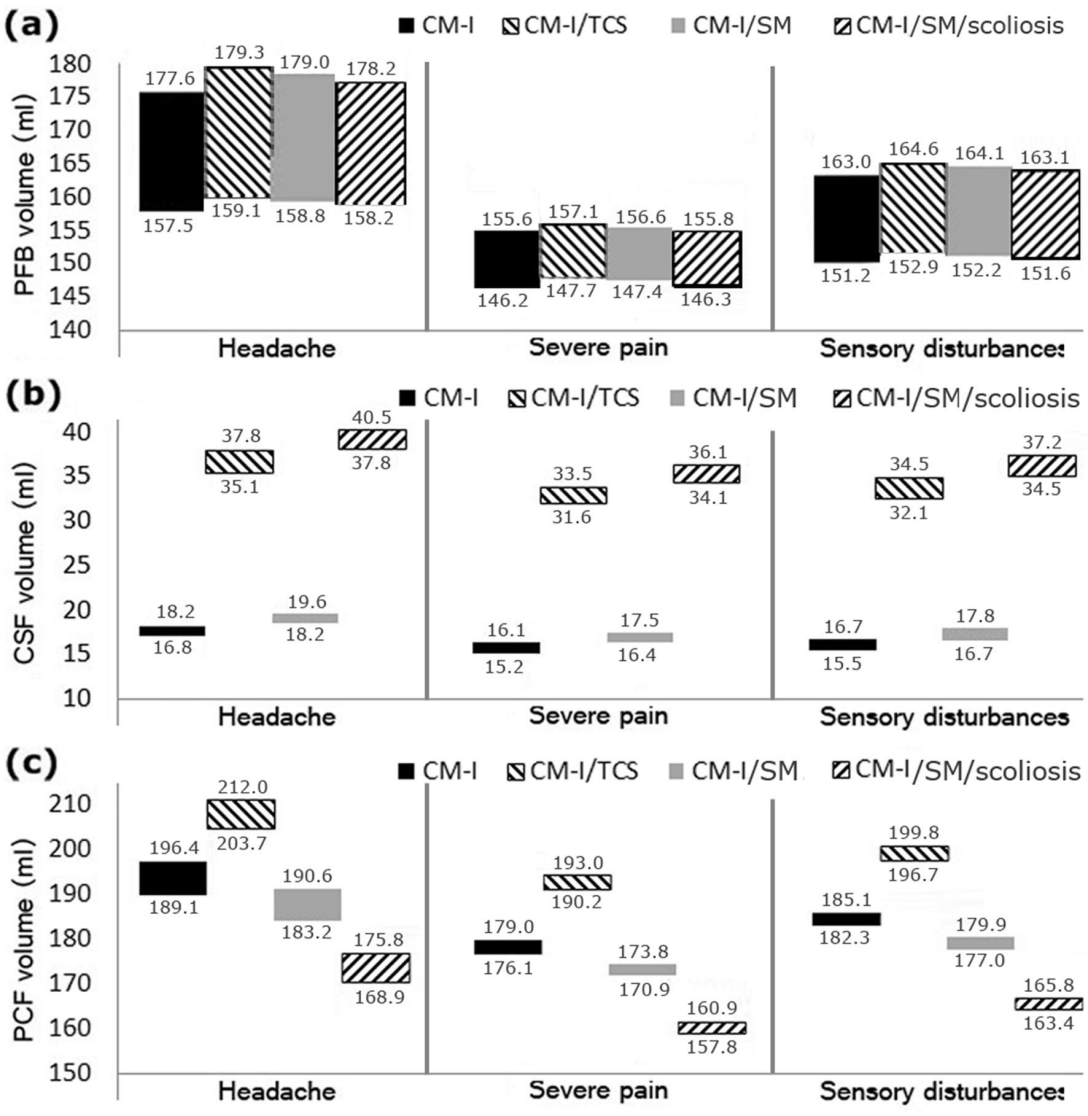
**(a)–(c)** show the relationship between prevalent symptoms of patients with PFB volumes, CSF volumes, and PCF volumes, respectively. For example, the intensity of the “headache” symptom was higher and more obvious than that of the other symptoms in the patients.

At the final section, the relationship was investigated between the three most prevalent symptoms of the patients (headache, severe pain, and sensory disturbances) and three volumetric parameters. The results of Fig. 6 showed that the mean values of PFB, CSF, and PCF volumes in patients with the headache symptom were about 11.1%, 8.4% and 11.3% more than those in patients with severe pain symptom, respectively (Fig. 6). Hence, in patients that the headache symptom was more obvious than other symptoms, the amounts of all the three volumetric parameters, especially PCF volume, were more than those in other patients. However, in patients with more obvious severe pain symptom compared to other symptoms, the amounts of all the three volumetric parameters, especially PCF volume, were less than those in other patients. Thus, there was a direct relationship between the patients’ symptoms and their volumetric parameters. According to Fig. 6, the range of volume changes for each symptom was separated merely for a quite limited PCF volume and this range was not separated sufficiently for the other two volumetric parameters. Therefore, these results, similar to the evaluation results of the correlation between the maximum CSF pressure and the volumetric parameters, showed that PCF volume was a more accurate and relevant volumetric parameter for assessing the conditions of these patients.

The deformable boundaries between CSF and the spinal subarachnoid space were neglected during the present biomechanical simulation as similar to the previous studies^21,43,45,46,53–56,72,73,85^. However, according to recent findings of Tangen et al.^70^, simulations using moving boundary might alter the observed maximum CSF pressure. Thus, comparative simulation using the FSI method is suggested for future studies. CSF was considered an incompressible Newtonian fluid flow in the present study, as in many previous studies^21,54,55,72,73^. This assumption is not unreal according to the previously mentioned studies, and it is probably sufficient for reaching acceptable results. However, the exact assessment of this assumption needs further studies. Furthermore, due to the time-consuming process of computer simulation, it was not possible to increase samples in each group. Future studies are suggested to investigate CM-I with other associations and recruit more patients to confirm the findings of the present study.

## Conclusion

Among the CSF hydrodynamic parameters, the maximum CSF pressure is a more accurate and relevant index for assessing conditions of CM-I patients. The results of this study suggest this index as a new hydrodynamic parameter for evaluating these patients’ conditions. The maximum CSF pressure was similar in patients with similar symptoms regardless of the group to which they belonged. Moreover, the results showed that PCF volume had a higher correlation than the other volumetric parameters with the maximum CSF pressure. Furthermore, among the volumetric parameters, PCF volume was more relevant for assessing the symptoms of these patients. In patients that the headache symptom was more obvious than other symptoms, all the volumetric parameters, especially PCF volume, had a higher amount compared to the other patients.

## Data Availability

For access the MRI files of subjects can contact to corresponding author.
